# Automated analysis method to assess pulmonary blood flow distribution using conventional X-ray angiography

**DOI:** 10.1038/s41598-022-18627-5

**Published:** 2022-08-22

**Authors:** Takuya Sakaguchi, Yuichiro Watanabe, Masashi Hirose, Kohta Takei, Satoshi Yasukochi

**Affiliations:** 1 Canon Medical Systems Corporation, Otawara, Tochigi Japan; 2 Department of Pediatric Cardiology, Nagano Children’s Hospital, Azumino, Nagano Japan; 3 Echo Center, Aizawa Hospital, Matsumoto, Nagano Japan

**Keywords:** Interventional cardiology, Biomedical engineering

## Abstract

Quantitative assessment of the right-to-left ratio of pulmonary blood flow distribution is important for determining the clinical indications for treating pulmonary arterial branch stenosis. A novel theory was recently proposed that can be used to quantitatively assess the right-to-left ratio on conventional X-ray angiography images. In the proposal, further developments were indicated, especially automated calculation. In this study, a new automated algorithm was developed. In the X-ray image, regions of interest were set in right and left lung, and time-signal intensity curves were measured. The new automated algorithm is applied to determine the optimal time window for the analysis of the time-signal intensity curve and to calculate the slope of the curve in the optimized time window. The right-to-left ratios in seven consecutive patients calculated by the new automated algorithm were compared to those calculated by lung perfusion scintigraphy. The ratios were in good agreement with linear regression with a slope of 1.27 and a Pearson correlation coefficient of 0.95. The processing time was less than 10 s, which is one-eighth of the manual processing time. The new automated algorithm is accurate, stable, and fast enough for clinical use in the real world.

## Introduction

Quantitative assessment of the right-to-left ratio of pulmonary blood flow distribution is important when determining the clinical indications for treating pulmonary arterial branch stenosis, which is often found in patients with congenital heart disease, such as tetralogy of Fallot and transposition of the great arteries, before and after surgical repair^1,2^. Semiquantifications are performed by lung perfusion scintigraphy (LS), and only qualitative observations are performed by conventional X-ray angiography (XA). Recently, a novel theory was proposed that can quantitatively assess the right-to-left ratio of pulmonary blood flow distribution using XA in the clinical setting^3^. This method uses a mathematical tracer kinetic model^4–8^ and measures the net increase in the time-signal intensity curves (TICs) in each right and left lung region of interest (ROI). This approach shows good correlation with LS with a Pearson correlation coefficient of 0.91 and a slope of linear fit of 1.2. Despite its promising results, this approach^3^ requires manual operation and needs some skills to obtain stable results because of manual variation. In addition, quick computational time is required during interventional procedures. Based on these requirements to avoid operator-dependent errors and to improve workflow with shortened measurement times, the goal of this study is to develop a new automated method to assess the right-to-left ratio of pulmonary blood flow distribution and compare this approach to lung perfusion scintigraphy.

## Algorithm

**Figure 1:**
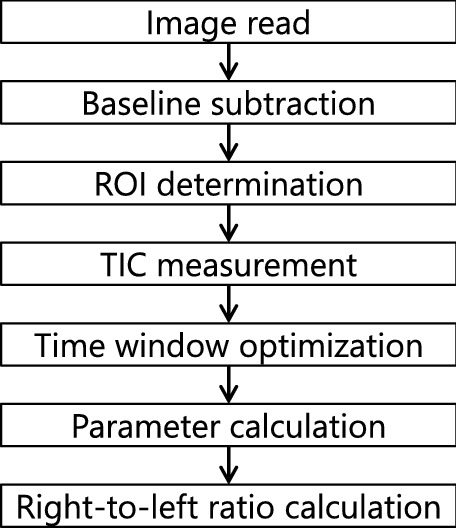
Image analysis process flow for right-to-left ratio of blood flow distribution.

**Figure 2:**
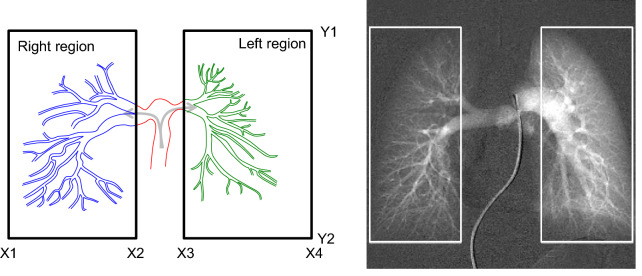
Regions of interest to measure lung right and left blood flow.

An overview of the proposed process flow is shown in Fig. 1. Contrast-enhanced X-ray pulmonary angiography images were acquired. The acquired images are incorporated in the image processing flow. The baseline mask image, obtained before contrast agent injection, is subtracted from subsequent, consecutive images. The ROI was determined in each right and left lung region, as shown in Fig. 2. The TIC of two ROIs are obtained. The temporal time window for the analysis of the obtained TIC is optimized by the new algorithm. The parameters of the TIC are calculated within each region for the optimized time window. Finally, the right-to-left ratio is calculated.

To achieve objective, quantitative, and reproducible automated methods, several key features are needed. These include accuracy, reproducibility, broad application to different types of diseases and quick computation that is clinically acceptable for the procedure. To address these requirements, in this work, we developed algorithm for determining ROI size, ROI location, automated optimization of the temporal time window, stable selection of parameters in the TIC, and minimization of computational time.

### ROI determination

For quantitative assessment of the right-to-left ratio of pulmonary blood flow distribution, rectangular ROIs are placed in the right and left regions, as demonstrated in Fig. 2. When an image is acquired, the field of view and source imager distance are usually adjusted so that the whole lung is maximally included in the image to minimize the patient radiation dose. Therefore, the ROI can be enlarged to cover the left and right lungs; these ROIs are close to the vicinity of the image edges, as shown in Fig. 2. The gap between the right ROI and left ROI at the middle of the image is increased as much as possible so that the main pulmonary trunk and tip of the catheter are excluded, but the whole lung region is included. A larger gap between the left and right ROIs is also beneficial when assessing many complex pediatric treatments, such as Blalock-Taussig shunts. In this study, ROI size and location are fixed for all cases analyzed. In 1024 by 1024 images, the ROI width is 350, and the ROI height is 820. The coordinates are shown in Fig. 2; right ROI (X1, Y1, X2, Y2) = (9, 103, 358, 922) and left ROI (X3, Y1, X4, Y2) = (665, 103, 1014, 922). ROI selection is not impacted by dynamic acquisition because diaphragm motion is not critical during a short period of time within 200 ms. The X-ray image acquisition angle of cranial (CRA) and caudal (CAU) directions can be applied as well as anterior–posterior (AP) directions. However, left anterior oblique (LAO) and/or right anterior oblique (RAO) directions cannot be used.

### TIC measurement

Contrast-enhanced XA images are incorporated in image processing. The baseline mask image, obtained before contrast agent injection, is subtracted from subsequent, consecutive images. The TIC of two ROIs were obtained by averaging all pixel values in each ROI. Using this averaging approach to calculate TIC, computational time is dramatically reduced. The original calculation requires image-based processing of all pixels in the image, which corresponds to image width by image height (for example, 1024 by 1024 pixels). However, the current ROI-based processing approach requires only two calculations (left and right ROI).

### Time window optimization

The right-to-left ratio of pulmonary blood flow distribution is calculated only in the specific temporal time window to measure equivalent blood flow with LS that has different tracer kinetic models^3,9,10^. In X-ray angiography, the temporal time window is required to be set at the torrent period during the second cardiac cycle after contrast injection. The torrent period is a short period during which the contrast agent is torrentially discharged from the pulmonary arteries to the capillary bed. The second cardiac cycle is used to eliminate variance in contrast agent concentration because contrast agent is not well mixed and unilaterally distributed in the pulmonary trunk in the first cardiac cycle immediately after contrast injection. This unilateral distribution leads to one side flow in the first cardiac cycle. Using the second cardiac cycle, this variance is reduced, and stable measurement is achieved.

In this paper, the mean TIC combining both the right and left regions is used, and the time of maximum slope of the combined TIC is detected. If one side flow occurred due to unilateral distribution in the pulmonary trunk, the combined TIC would have a small slope because the total amount of contrast flow was small; hence, the time of one side flow would not be detected. If the contrast is well mixed, the contrast agent flows to both the right and left regions simultaneously, the total amount of contrast flow is large, and the combined time-signal density curve should have a steep slope.

The length of the time window is set to less than 200 ms; six frames in the case of 30 frames/s data acquisition. This is because the period from the time when contrast agent arrives at the first branch of the pulmonary artery to the time when contrast agent fills the entire lung field is approximately 200 ms.

**Figure 3:**
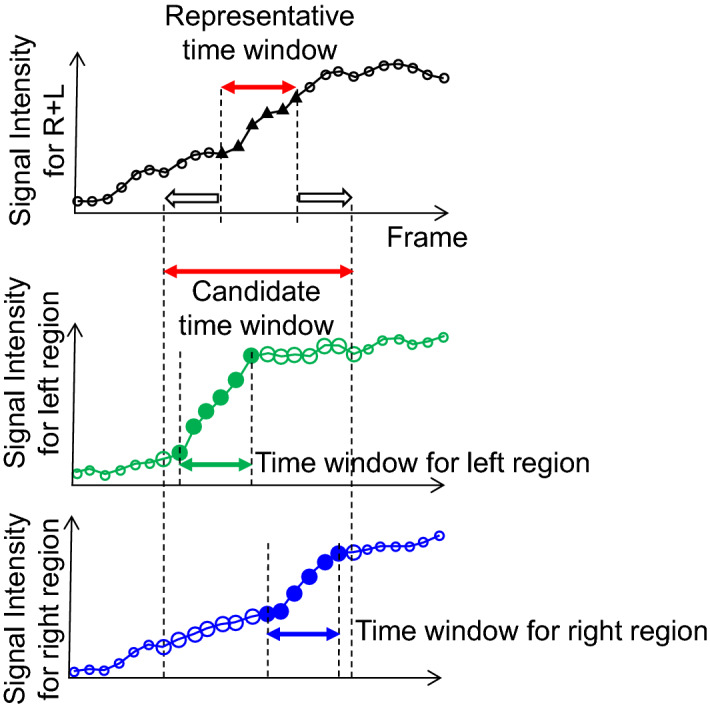
New algorithm to optimize time window to measure pulmonary blood flow distribution.

We observed that the starting time of contrast flow from the pulmonary trunk was slightly different between the right and left sides. The difference is up to 100 ms. This difference does not affect LS measurements that count temporally accumulated tracer^11,12^. On the other hand, it impacts the proposed method because the proposed method does not measure accumulation but measures the net increase in TIC in a short time window. In this paper, a new automated algorithm is proposed to achieve stable results even in cases when the contrast flow starting time is slightly different. In this algorithm, the time window is optimized for the right and left lung regions independently. First, a representative six-frame time window is detected by using the above combined TIC. Second, it is extended by eight frames: four frames before and after the representative six frames. A total of 14 frame lengths are determined as a candidate time window. Third, in this 14 candidate frame time window, six frames that show the maximum slope of the TIC are selected in each right and left region independently. These steps are shown in Fig. 3. In summary, optimized time windows are selected for each right and left region independently in the same cardiac cycle.

### Parameter calculation

The right-to-left ratio of pulmonary blood flow distribution is calculated by the net increase in signal intensity and is an equivalent model with scintigraphy^3^. In this paper, a stable selection of parameters is investigated. If only two points are used to measure net increase in signal intensity, it is easily affected by several noise factors, such as body motion, heart motion, and image acquisition noise. Therefore, in this paper, all six points in the time window are used to calculate the slope using linear fitting. This approach is equivalent to the scintigraphy method, and it makes the algorithm stable and robust.

## Evaluation methods

Before testing our kinetic model on patients, approval was obtained from the Institutional Review Board and ethical committee of Nagano Children’s Hospital (approval number IRB-28-1). All methods were performed in accordance with the relevant guidelines and regulations. After obtaining written informed consent from pediatric patients’ parents, 11 consecutive subjects with congenital heart disease were enrolled in this pilot study and underwent XA and LS between September and November 2016. Patients whose pulmonary blood flow was supplied by multiple vessels, patients who had extra blood supply in addition to the main pulmonary artery, patients who had lacked imaging of the lung field, and patients who had overlapping images of the main pulmonary artery were excluded. Of the 11 initial patients, seven who met the inclusion criteria were analyzed.

LS was performed using an e.cam with an e.soft workstation (Canon Medical Systems Corporation, Japan) using ^99m^Tc-MAA (radionuclide) as a radioisotope tracer. Planar images of both lungs in six directions, including the anterior–posterior (AP) and posterior-anterior (PA) directions, covering the entire lung field were acquired with a LEHR collimator. The counts of each lung were averaged from both the AP and PA images. The counts were then converted to radioisotope tracer volumes using a predetermined calibration factor to obtain quantitative pulmonary blood flow.

XA was performed using a cardiovascular X-ray imaging system (Canon Medical Systems Corporation, Japan) within 3 days before or after LS. The imaging parameters were as follows: field of view 5–8 in., fixed tube voltage, pulse rate 30 frames per second, image matrix size 1024 by 1024, and no automatic brightness control or nonlinear image postprocessing. The total acquisition time was 6–10 s. Iodine contrast agent (Iopaque 300, Fuji Pharma, Japan) was injected as a bolus (1 ml/kg/second) into the pulmonary trunk through a 4–6 Fr catheter. The images were acquired continuously starting one second prior to contrast injection until all contrast agent was washed out from the lung field to the descending aorta on the AP projection. All images were stored in a workstation in DICOM format. The images were automatically analyzed by in-house software using the data processing protocol described in this paper. Manual analysis was performed as described in a previous paper^3^.

**Figure 4:**
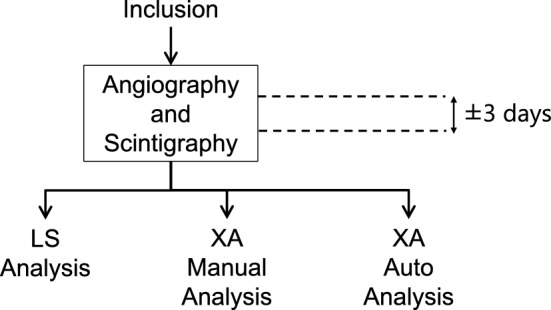
Evaluation method to compare the XA result with the LS result.

Angiography analysis results were compared with LS. The evaluation comparison method is described in Fig. 4. The data analysis was performed using ImageJ (NIH, USA) and Microsoft Excel. Statistical analysis was performed using R version 4.1.0 (R Foundation for Statistical Computing, Vienna, Austria). The in-house software code used MATLAB R2017b (MathWorks, USA).

## Results

**Figure 5:**
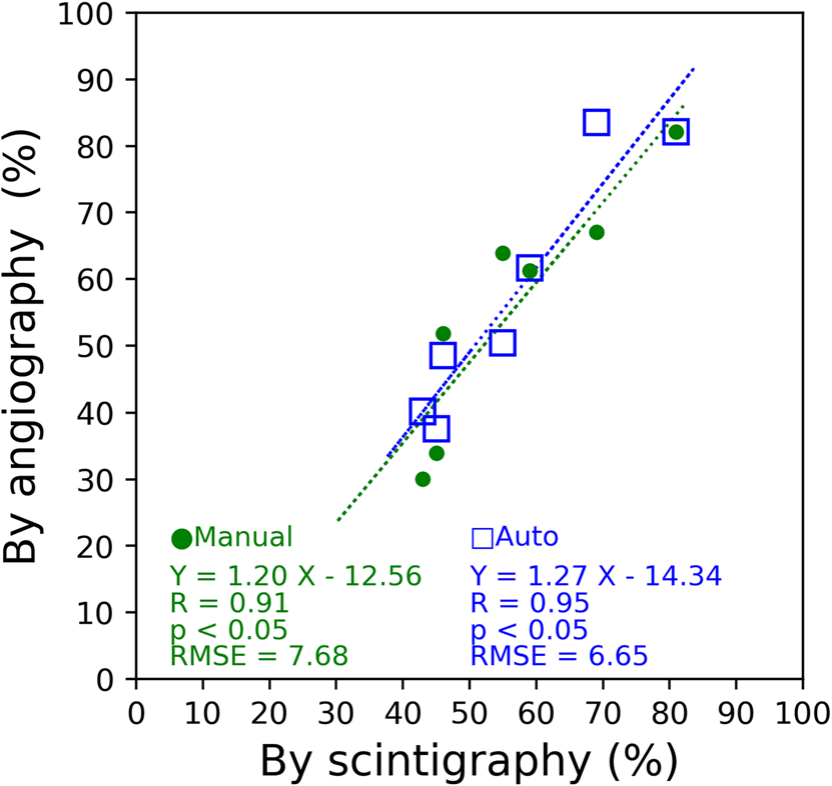
The ratio of right pulmonary blood flow distribution. The blue rectangle is the XA auto to LS result. The green circle is the XA manual to the LS result^3^.

**Figure 6:**
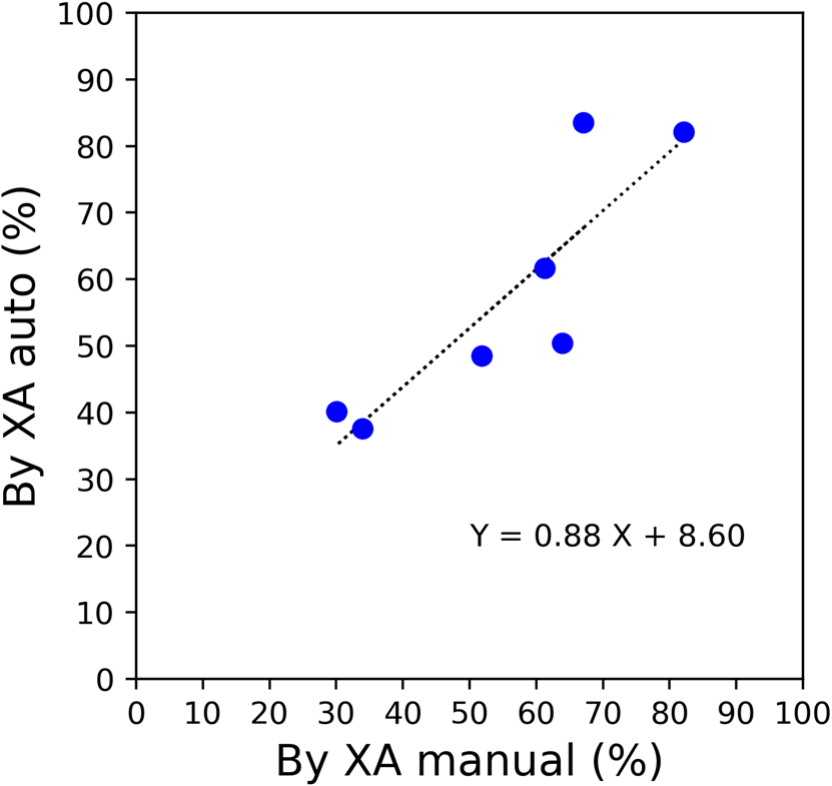
Comparison of the ratio of right pulmonary blood flow distribution between the XA manual and XA auto.

A comparison of the right-to-left ratio results among LS, XA manual^3^, and XA auto is shown in Table 1, Figs. 5 and 6. The linear fit of XA auto to LS has a slope of 1.27, root mean square error (RMSE) of 6.65, and a Pearson correlation coefficient of 0.95 (p < 0.05). The mean difference in the time window between right and left is 0.7 frames, 0.02 s, or 5.1% of the R-R interval.

**Table 1:** Comparison of the right-to-left ratio among LS, XA manual, and XA auto.

Patient	Right-to-left ratio using LS	Right-to-left ratio using XA manual	Right-to-left ratio using XA auto	First point of time window for right region in XA auto, in frame number, elapsed time from injection, and cardiac phase	First point of time window for left region in XA auto, in frame number, elapsed time from injection, and cardiac phase
A	81:19	82:18	82:18	22, 0.73 s, 5%	22, 0.73 s, 5%
B	59:41	61:39	62:38	26, 0.86 s, 4%	26, 0.86 s, 4%
C	69:31	67:33	84:16	16, 0.53 s, 11%	16, 0.53 s, 11%
D	43:57	30:70	40:60	24, 0.79 s, 30%	21, 0.69 s, 8%
E	45:55	34:66	38:62	23, 0.76 s, 4%	23, 0.76 s, 4%
F	55:45	64:36	50:50	15, 0.50 s, 14%	14, 0.46 s, 7%
G	46:54	52:48	49:51	16, 0.53 s, 5%	15, 0.50 s, 98%

**Figure 7:**
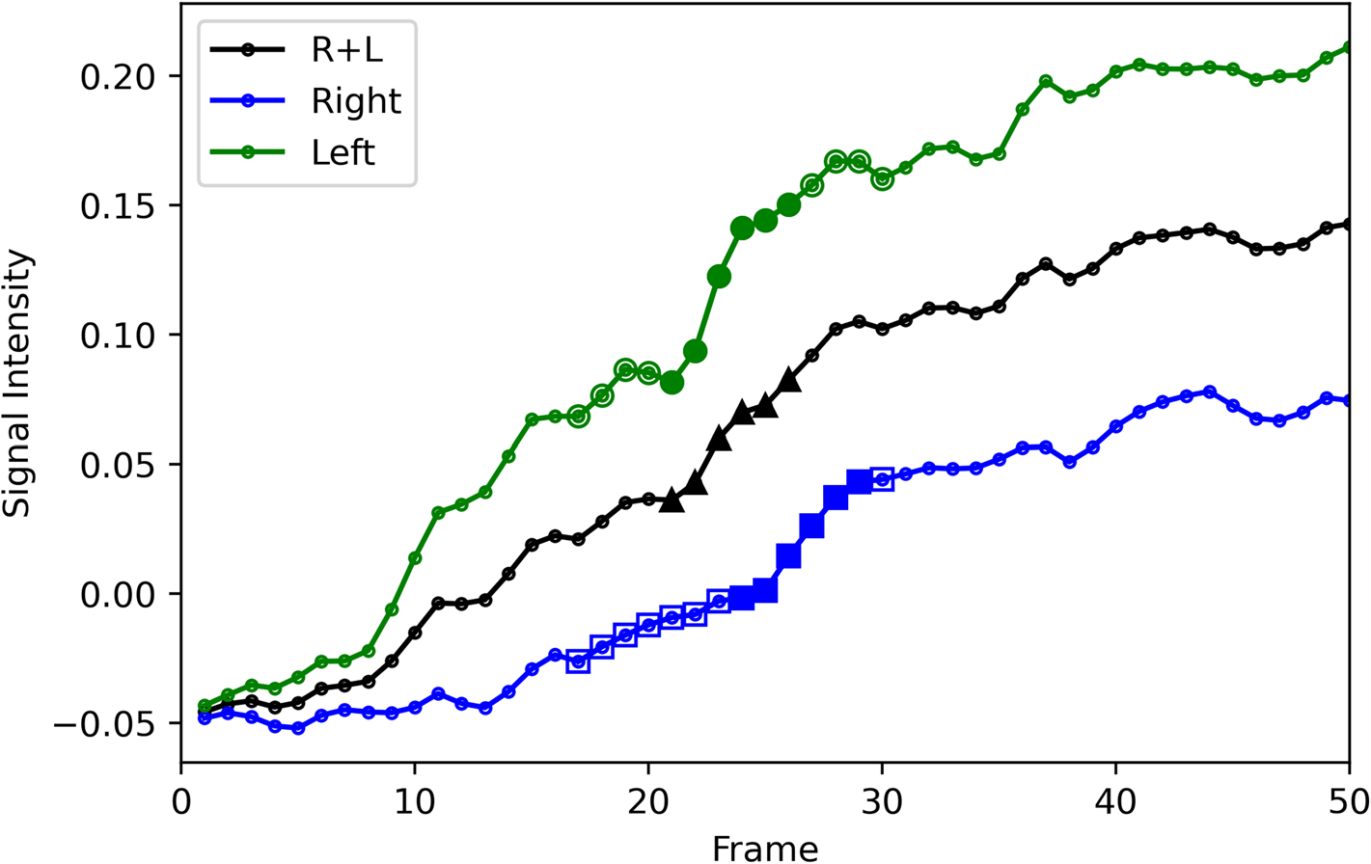
Example of a time-signal intensity curve. Blue represents the right lung region, green represents the left lung region, and black represents the mean of the right and left regions (R + L). The triangle mark is the representative time window using the R + L curve. Transparent circle and rectangle marks are candidate points. Filled circle and rectangle marks are the final optimized points.

**Figure 8:**
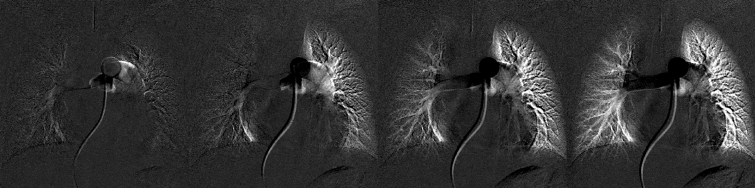
Corresponding image of Fig. 7, frame 22, 25, 28, and 31. For better visualization, frame 21 was subtracted from all the other frames. The starting time of blood flow supply was slightly different between the right and left lung regions.

An example of TIC for patient D is shown in Fig. 7 (Supplementary Table S1), and its corresponding image is shown in Fig. 8. The horizontal axis corresponds to the frame number, where frame #1 is the first frame after contrast injection. In this case, the starting times of the blood flow to the right and left lung regions were slightly different. The difference can be observed in the figure; the slope of the TIC of the right region is slightly shifted to the right compared to the left region. Using TIC combining both the right and left regions (R + L), a representative six-frame time window (frame 21–26) was detected where the maximum slope is observed, as shown with triangle marks. The time window is extended by six frames to 14 frames by including four additional frames in each side of the selected representative time window. In the selected 14 frame candidate time window (frames 17–30), each six frames that show maximum slopes of each right and left TIC are selected independently (frames 21–26 for the left region that are marked as filled circles and frames 24–29 for the right region that are marked as filled rectangles). The slope for the right region is 0.009824, and the slope for the left region is 0.014666. The right to left region was calculated as 40:60.

The overall processing time from start (software reading DICOM images) to end (display of final right-to-left ratio) is measured using an Intel Core i7-7700 CPU 3.60 GHz, RAM 16.0 GB, OS Microsoft Windows 10 Pro. The processing time by XA auto is nine seconds. The XA manual required processing time varied between subjects; its mean ± standard deviation was 73 ± 38 s.

## Discussion

An automated analysis method was developed to assess pulmonary blood flow distribution using conventional X-ray angiography. The accuracy was verified by comparing the results to lung perfusion scintigraphy, which resulted in a Pearson correlation coefficient of 0.95 (p < 0.05). All seven consecutive patients’ data were successfully analyzed. The processing time was nine seconds using a general purpose workstation. Several unique technical features were adopted in the processing. Lung regions were optimally set at each right and left region to include the whole lung region but exclude the pulmonary trunk and catheter. For automated time window optimization, the mean first point of the time window was 0.66 s after contrast injection. If the pediatric patient’s heart rate is assumed to be 120 beats/min, 0.66 s means that the first cardiac cycle after contrast injection is not used for analysis, and the second cycle is used. By using the second cycle, variance in contrast agent concentration can be eliminated; contrast agent is not well mixed and unilaterally distributed in the pulmonary trunk in the first cardiac cycle. The mean cardiac phase of the first point of the time window was at 7.6% of the R-R interval. This phase is at the torrent period in the systolic phase when the heart is contracted, and contrast agent is torrentially discharged from the pulmonary arteries to the capillary bed. This means that the proposed algorithm successfully optimized the time window for automatic analysis by using angiography to achieve an equivalent tracer kinetic model with scintigraphy. The mean difference in the starting time of contrast flow from the pulmonary trunk to the right and left lungs was 0.7 frames (0.02 s), and the maximum difference was three frames (0.1 s). This is a small difference, and the new algorithm could handle these cases. For parameter identification, the slope of the linear fitting of the six frames was calculated, and stable analysis was achieved. In summary, a good automated analysis method was established to assess pulmonary blood flow distribution using conventional X-ray angiography.

Assessment of the asymmetric distribution of pulmonary blood flow is important^2^. Its diagnosis is established by using imaging modalities such as scintigraphy, Magnetic Resonance Imaging (MRI), and Computed Tomography (CT)^9,10^. On the other hand, there are few studies performed by conventional angiography, and its assessment is limited to qualitative analysis^13,14^. Automated XA processing contributes significantly to patient treatment. First, assessment can be executed immediately after completion of treatment while the patient is in the catheter-laboratory and the patient does not need to move to the scintigraphy examination room. The proposed method can generate results by XA auto in nine seconds, which is significantly faster than 73 s by manual XA. The new approach enables interventionalists to obtain the result seamlessly after image acquisition without interruption of their interventional treatment operation. The proposed method uses conventional XA images that are usually acquired during the procedure, so additional image acquisition is not required for this purpose. This also reduces patient burden, especially in pediatric patients with congenital heart disease. Second, automation can introduce objective, quantitative, and reproducible results. Operator dependency is avoided during highly stressful interventional treatment, and the need for skilled operator training can be reduced. Hence, quantitative comparisons, such as pre- and postprocedure, have been achieved.

We note that there are some drawbacks of automation. For example, the operator may likely use the automatic feature only by reading the final ratio number without confirming the intermediate process result, which may help to avoid critical error or misusage. There is also a possibility that automated XA findings are suboptimal compared to manual XA when LS results are set as the gold standard. One of the biggest factors affecting automated XA accuracy is ROI size and location. In this study, ROI size and location are fixed. As a result, the right-to-left ratio may vary when the patient position is not at the center of the image or the pulmonary artery is not equally located in the right and left ROI. For future improvement, several approaches will be tested; for example, (a) the patient position is set to the center of the image at acquisition, (b) the acquired image is transformed so that the patient position is located at the center of the image, or (c) the ROI location is adjusted to be equally distributed to the right and left of the patient lung by image recognition, such as machine learning.

The limitation of this paper is that this is only an introduction into technical methodology. Real-world studies are strongly recommended to validate many types of diseases to determine whether automation can be performed for all cases. For example, the length of the time window may be adjusted for adult patients, heart rate, and image acquisition frame rate. Further optimizations are required for patient selection, image scan condition, frame rate, field of view, angulation, and image analysis parameters.

## Conclusions

An automated analysis method was developed and verified to assess pulmonary blood flow distribution using conventional X-ray angiography. The method is accurate, stable, and quick and can be used during interventional pulmonary treatment.

## Supplementary Information

Supplementary Table S1.

## Data Availability

The datasets generated and/or analyzed during the current study are not publicly available due to patient privacy but are available from the corresponding author on reasonable request.
